# HIV Risky Sexual Behaviors and HIV Infection Among Immigrants: A Cross-Sectional Study in Lisbon, Portugal

**DOI:** 10.3390/ijerph110808552

**Published:** 2014-08-20

**Authors:** Sónia Dias, Adilson Marques, Ana Gama, Maria O. Martins

**Affiliations:** 1 Instituto de Higiene e Medicina Tropical - Centro de Malária e Outras Doenças Tropicais, Universidade Nova de Lisboa, Rua da Junqueira n° 100, Lisboa 1349-008, Portugal; E-Mails: anafgama@gmail.com (A.G.); mrfom@ihmt.unl.pt (M.O.M.); 2 Faculdade de Motricidade Humana, Universidade de Lisboa, Estrada da Costa, Cruz Quebrada–Dafundo 1499-002, Portugal; E-Mail: amarques@fmh.ulisboa.pt

**Keywords:** immigrants, HIV infection, risky sexual behavior

## Abstract

This study aimed to examine risky sexual behavior, its associated factors and HIV infection among immigrants. A participatory cross-sectional survey was conducted with 1187 immigrants at the National Immigrant Support Centre, in Lisbon (52.2% female; 34.0% Africans, 33.8% Brazilians, 32.2% Eastern Europeans). About 38% of participants reported ≥2 sexual partners in the previous year, 16.2% both regular and occasional sexual partners (last 12 months), 33.1% inconsistent condom use with occasional partners, and 64% no condom use in the last sexual intercourse. Unprotected sex in the last sexual intercourse was more likely among women, Africans, those older, with elementary education, those married and those who didn’t receive free condoms in the previous year. No condom use was less likely among those having only occasional sexual partners and both regular and occasional sexual partners. One third of participants had never been tested for HIV. Those never tested reported more frequently inconsistent condom use than those ever tested. Overall, 2.0% reported being HIV positive (2.5% of men; 4.4% of Africans); 4.3% admitted having a STI in previous year. HIV-positive immigrants reported high-risk sexual behaviors. Tailored interventions to promote awareness of HIV serostatus among immigrants as well as culturally adapted risk reduction strategies should be strengthened.

## 1. Introduction

The HIV epidemic continues to be a major public health concern in the European Union [1,2]. Increased international migration, particularly from highly endemic countries, has been acknowledged as one of the major factors influencing the epidemiology of HIV in Europe and contributing to the changing pattern of HIV transmission in this region, where in the recent years sexually transmitted cases have been on the rise [3]. Estimates indicate that approximately 40% of the HIV diagnosed cases during 2007–2011 in Europe were in migrants [1].

Migration had been recognized to be associated with increased high risk sexual behaviors [4,5]. Previous research indicate that a considerable amount of immigrants report to have multiple partners and to not use condom consistently with both occasional (casual partner with no committed relationship) and regular (main, stable partner) partners [6,7,8,9], which renders this population particularly at risk for HIV infection. Explanations of the increased vulnerability of immigrants to HIV infection include the long periods away from family and partners, the associated need to seek companionship to compensate for the alienating aspects of the migration experience and the fewer social controls on behavior [9,10], but also the experience of social exclusion and lack of legal protection that often translate in lesser means of protection and poorer health status [7,9], and barriers to health services (related to their legal status, socioeconomic conditions, language and cultural difference) [3,11,12,13].

Despite the evidence of disproportionate HIV risk among migrants and increasing numbers of immigrants in the European context, there are few studies aimed to examine the extent of HIV infection, risky sexual behavior and its determinants among these populations. Additionally, many immigrants have not even been systematically included in HIV surveillance systems, as undocumented and recent immigrants who are omitted in the population census and records. Also, many groups underuse health services where surveillance data is reported (mainly due to mistrust or lack of knowledge about services available and migrants’ health rights). Furthermore, in many cases data are not collected in a way that allows disaggregated analysis (in many countries no data is collected on variables as country of birth or ethnic group) [14,15].

Knowledge on HIV infection and risk behavior among immigrants is greatly needed to inform HIV prevention and control national programs as well as to provide valuable evidence for appropriate interventions in this population. This is particularly relevant in Portugal as it is one of the countries presenting the largest burden of infection in Western Europe-data estimates that in 2011 it had one of the highest incidence rates (11.8 per 100,000 population; 17.2 per 100,000 males and 6.7 per 100,000 females) and the second highest HIV prevalence (0.7% among adults aged 15–49 years old) in the European Region [16,17]. Also, in the last decades Portugal has received many immigrants (the foreign-born population represent 8.3% of the total population in Portugal), mainly from countries with high HIV prevalence as most Portuguese-speaking African countries, Eastern European countries and Brazil [17,18,19]. In 2012, 29.4% (228) of the newly diagnosed cases in Portugal referred to immigrants: 66.4% from Sub-Saharan Africa, 20.0% from Latin America, 11.4% from Europe [20]. This study aimed to examine risky sexual behaviors, its associated factors and HIV infection among immigrants in the Lisbon Metropolitan Area.

## 2. Methods

### 2.1. Study Design and Population

A cross-sectional survey was conducted with immigrants in the Lisbon Metropolitan Area. This area has currently the highest concentration of immigrant population in the country. Official data indicate that, in 2011, 44% of the immigrant population in Portugal resided in the Lisbon region (around 188,259 and an undetermined number of undocumented persons excluded in the official statistics) [21]. A participatory approach was used: representatives of governmental organizations (public health services), non-governmental organizations (NGOs) and associations of African, Brazilian and Eastern European immigrants (who work in outreach projects in the areas of health promotion, HIV prevention and social support with immigrant communities) actively collaborated in all phases of the study.

### 2.2. Sampling and Data Collection

The study sample included 1187 immigrants who were interviewed at the National Immigrant Support Centre (NISC) in Lisbon over a 1 month period (February) in 2011. The NISC is part of the High Commission for Immigration and Intercultural Dialogue. Within a friendly environment, it provides integrated answers to needs faced by immigrants residing in Portugal (e.g., regularization process, interaction with public services) regardless of legal status or any other criterion. It brings together in one place different public institutions in the areas of health, education, social security, employment and justice [22,23].

All immigrants who visited the NISC premises during working hours were approached and invited to participate. The inclusion criteria were being an immigrant, defined as a non-national person who migrated to Portugal for the purpose of settlement [24], and being ≥18 years old. The proportion of refusals was 9.7% (*n* = 127; 57.7% were female). No further information was collected from those who refused.

Data were collected through an anonymous structured questionnaire. Given the sensitive nature of the subject under investigation, the interviews took place in a quiet and isolated room at NISC offices to ensure privacy and comfort of participants. The questionnaire was applied by trained interviewers from immigrant communities, recruited and selected in collaboration with NGOs and immigrant associations. Interviews were applied in Portuguese—it is the official language of Africans and Brazilians in Portugal and many Eastern Europeans are fluent in Portuguese. In the few cases of Eastern Europeans who weren’t fluent in Portuguese, specific interviewers applied the questionnaire in the native language. The questionnaire was administered using pen and paper. The interviewers training included information about the questionnaire, the data collection procedures and general interview techniques.

Anonymous participation and confidentiality of data was guaranteed. Informed consent was obtained. The study was approved by the Ethical Committee of Instituto de Higiene e Medicina Tropical, Universidade Nova de Lisboa.

### 2.3. Instrument

The questionnaire comprised items on sociodemographic characteristics, sexual behaviors, HIV testing, self-reported HIV infection and other STI, use of health services and prevention initiatives. In more detail:

*Sociodemographic characteristics*: These included sex, age (continuous variable), educational level (“elementary”, “secondary”, “higher”), marital status (“single”, “married”, “divorced or separated” and “widowed”; for analysis these options were dichotomized into “unmarried”—single/divorced or separated/widowed—and “married”), professional situation (“employed part-time”, “employed full-time”, “working-student”, “student”, “unemployed” and “retired”; for analysis these options were dichotomized into “non-employed”—student/unemployed/retired–and “employed”-employed part-time/employed full-time/working-student), perceived monthly income (“very insufficient”, “insufficient”, “sufficient” and “more than sufficient”; for analysis these options were dichotomized into “insufficient” and “sufficient”), and migration-related characteristics such as origin (response options included a list of countries which were then aggregated into “African”, “Brazilian” and “Eastern European”), length of stay (in months for those residing in Portugal for <1 year, and in years for those residing for ≥1 year) and immigration status (“undocumented”, “documented”).

*Sexual behaviors*: Participants were asked about age at first sexual intercourse (in years); engagement in sexual intercourse (yes/no), number and type of sexual partners, commercial sex (yes/no), sex with a same-sex partner (yes/no) and condom use by type of sexual partner in the reference period of 12 months; type of sexual partner and condom use in the last sexual intercourse. Regarding type of sexual partner, two response options were used (regular/occasional partner)—regular sexual partner was defined as a person with whom one has sexual intercourse and has plans for further long term or committed relationship; occasional sexual partner was defined as a person with whom one has sexual intercourse (without payment) and has no plans for further long term or committed relationship [8]. As these response options were not mutually exclusive, for the analysis they were categorized into “only regular”, “only occasional” and “regular and occasional”. Engagement in commercial sex was defined as having had sexual intercourse with a person involving money or goods exchange between the two parties [25]. Regarding condom use in the last 12 months, participants were asked how often a condom was used (by him/herself or his/her sexual partner) during their sexual relations, having three possible response options (always/sometimes/rarely or never). For analysis this variable was dichotomized into consistent (always) and inconsistent (sometimes/rarely or never) condom use. Participants were also asked whether a condom was used (by him/herself or his/her sexual partner) in their last sexual intercourse, being the possible response options “yes” and “no”.

*HIV testing*: Participants provided information on whether they had been tested for HIV ever and in the previous 12 months, both questions with “yes”/“no” response options.

*HIV/STI status*: Participants were asked to self-report their current serostatus for HIV and their serostatus for other sexually transmitted infections (STIs) in the last 12 months.

*Use of health services and prevention initiatives*: Participants were asked about ever use of health services in Portugal, ever attendance of a sexual and reproductive health consultation and having received free condoms in the previous 12 months, all with “yes”/“no” response options.

The questionnaire was designed in order to provide data for the second generation indicators created in accordance with international guidelines [26,27]. The items used in the questionnaire were also based on other international HIV research projects on migrants [28,29,30]. The instrument was constructed along with feedback provided by the partners of the study. The questionnaire was pre-tested with members of immigrant communities; few amendments were made to improve clarity and appropriateness of the questions to the study populations.

### 2.4. Data Analysis

Data analysis was performed using IBM SPSS Statistics v.22. The bivariate associations between sociodemographic characteristics, migration-related variables, sexual behaviors, HIV testing, self-reported infection, use of health services, prevention initiatives and gender were assessed using χ^2^ and Fisher’s exact tests when appropriate. Logistic regression analyses were performed to estimate the crude odds ratios (OR), adjusted OR and 95% confidence intervals (CI) of factors associated with no condom use in the last sexual intercourse. For analysis of sexual behaviors in the previous 12 months, 176 participants were excluded as they haven”t reported sexual intercourse during this period.

## 3. Results

### 3.1. Characteristics of Participants

The socio-demographic and migration-related characteristics of the male and female participants are shown in Table 1. In brief, 52.2% of the sample was female. Approximately two thirds of the participants were 25–44 years old. Overall, 34% were Africans (from Portuguese-speaking African countries: Angola, Cape Verde, Guinea Bissau, Mozambique, and Sao Tome and Principe), 33.8% were Brazilians and 32.2% Eastern Europeans (from Ukraine, Moldavia, Russia, Romania, Belarus and Bulgaria). About 38% had secondary education, more men than women (*p* = 0.028). More than a half of participants were not married. A third reported being undocumented, 47.1% were non-employed and 57.6% perceived their monthly income as insufficient. Mean length of stay in Portugal was 7.5 years (±7.5).

**Table 1:** Socio-demographic and migration-related characteristics by gender.

	Total		Men		Women		*p ^a^*
	n	%	n	%	n	%	
Total	1187	100	567	47.8	620	52.2	
Age (years) (*n* = 1187)							0.173
18–24	189	15.9	101	17.8	88	14.2	
25–44	751	63.3	356	62.8	395	63.7	
45–65	247	20.8	110	19.4	137	22.1	
Origin (*n* = 1187)							0.997
Eastern European	383	32.2	183	32.3	200	32.2	
Brazilian	401	33.8	191	33.7	210	33.9	
African	403	34.0	193	34.0	210	33.9	
Educational level (*n* = 1182)							0.028
Higher	419	35.4	187	33.2	232	37.5	
Secondary	448	37.9	236	41.8	212	34.3	
Elementary	315	26.6	141	25.0	174	28.2	
Marital status (*n* = 1184)							0.457
Unmarried	723	61.1	340	60.0	383	62.1	
Married	461	38.9	227	40.0	234	37.9	
Immigration status (*n* = 1176)							0.084
Undocumented	390	33.2	200	35.7	190	30.9	
Documented	786	66.8	361	64.3	425	69.1	
Professional status (*n* = 1187)							0.905
Non-employed	559	47.1	266	46.9	293	47.3	
Employed	628	52.9	301	53.1	327	52.7	
Perceived monthly income (*n* = 1184)							0.904
Insufficient	682	57.6	325	57.4	357	57.8	
Sufficient	502	42.4	241	42.6	261	42.2	
	**Mean ± SD**		**Mean ± SD**		**Mean ± SD**		***p*^b^**
Length of stay in Portugal (years) (*n* = 1181)	7.5 ± 7.5		7.8 ± 7.9		7.2 ± 7.1		0.200

^a^ Tested by Chi-square or Exact Fisher Test; ^b^ Tested by T-test.

### 3.2. Risk Sexual Behaviors, HIV Testing and HIV Infection

Table 2 presents the data on risk sexual behaviors, HIV testing and reported HIV infection of the participants stratified by gender. Compared to women, men reported lower mean age at first sexual intercourse and higher number of sexual partners in the last year. Also, a higher proportion of men reported having both regular and occasional sexual partners in the last year and having their most recent sexual intercourse with an occasional partner. Also, a higher proportion of men reported engagement in sexual intercourse with same-sex partners and in commercial sex in the previous year, comparing to women.

Condom use with occasional partners in the last 12 months was inconsistent among 33.1% of participants, significantly more women than men (44.8% and 28.7%, respectively). Inconsistent condom use with regular partners was reported by 81.7% of participants, with no significant differences across gender. About 73% of women and 54.2% of men didn’t use condom at their most recent sexual intercourse.

Approximately 38% of participants reported having never been tested for HIV, more men than women; of those ever tested, 63.4% were not tested in the last year. Those never tested reported more frequently inconsistent condom use with occasional partners (42.7% compared to 28.9% of those ever tested for HIV, *p* = 0.034) and with regular partners (85.2% *vs.* 79.8% of those ever tested for HIV, *p* = 0.05).

**Table 2:** Risk sexual behaviors, HIV testing, reported HIV infection, use of health services and prevention initiatives by gender.

	Total		Men		Women		
	Mean ± SD		Mean ± SD		Mean ± SD		*p* ^a^
Age at first sexual intercourse (*n* = 975)	16.9 ± 2.9		16.2 ± 2.9		17.5 ± 2.8		<0.001
	**n**	**%**	**n**	**%**	**n**	**%**	***p*^b^**
Number of sexual partners in the previous 12 months (*n* = 974)							<0.001
1 partner	604	62.0	233	50.0	371	73.0	
2–3 partners	256	26.3	147	31.5	109	21.5	
≥ 4 partners	114	11.7	86	18.5	28	5.5	
Type of sexual partners in the previous 12 months (*n* = 998)							<0.001
Only regular	737	73.8	284	59.9	453	86.5	
Only occasional	99	9.9	77	16.2	22	4.2	
Regular and occasional	162	16.2	113	23.8	49	9.4	
Sexual intercourse with same-sex partners in the previous 12 months (*n* = 1002)							0.007
No	943	94.1	437	92.0	506	96.0	
Yes	59	5.9	38	8.0	21	4.0	
Commercial sex in the previous 12 months (*n* = 1011)							0.002
No	972	96.1	450	94.1	522	97.9	
Yes	39	3.9	28	5.9	11	2.1	
Type of sexual partner at the most recent sexual intercourse (*n* = 1008)							<0.001
Regular	827	82.0	348	73.0	479	90.2	
Occasional	181	18.0	129	27.0	52	9.8	
Condom use with regular partners in the previous 12 months (*n* = 890)							0.068
Consistent	163	18.3	83	21.0	80	16.2	
Inconsistent	727	81.7	313	79.0	414	83.8	
Condom use with occasional partners in the previous 12 months (*n* = 248)							0.017
Consistent	166	66.9	129	71.3	37	55.2	
Inconsistent	82	33.1	52	28.7	40	44.8	
Condom use in the last sexual encounter (*n* = 992)							<0.001
No	635	64.0	254	54.2	381	72.8	
Yes	357	36.0	215	45.8	142	27.2	
Ever testing for HIV (*n* = 1185)							<0.001
Yes	740	62.4	316	55.8	424	68.5	
No	445	37.6	250	44.2	195	31.5	
Reported HIV status (*n* = 735) ^1^							0.407
Negative	720	97.3	307	97.5	413	98.3	
Positive	15	2.0	8	2.5	7	1.7	
Ever utilization of health services in Portugal (*n* = 1186)							<0.001
No	207	17.5	125	22.0	82	13.2	
Yes	979	82.5	442	78.0	537	86.8	
Attendance to a sexual and reproductive consultation (*n* = 1169)							<0.001
No	876	74.9	489	87.6	387	63.3	
Yes	293	25.1	69	12.4	224	36.7	
Free condoms received in the previous 12 months (*n* = 1186)							0.047
Yes	391	33.0	203	35.8	188	30.4	
No	795	67.0	364	64.2	431	69.6	

^1^ For the analyses it was only considered participants who reported to have been tested for HIV; ^a^ Tested by T-test; ^b^ Tested by Chi-square or Exact Fisher Test.

Of those ever tested for HIV, 15 participants (2.0%) reported being HIV positive: 2.5% of men and 1.7% of women; 4.4% (*n* = 12) of Africans, 1.2% (*n* = 2) of Eastern Europeans and 0.3% (*n* = 1) of Brazilians (data not shown in the table). HIV-positive participants reported high-risk sexual behaviors: nine participants (60.0%) had two or more sexual partners in the last year, six (40.0%) had occasional sexual partners and three (20.0%) both regular and occasional sexual partners in the last year, four (26.7%) inconsistently used condom with occasional partners and four (26.7%) with regular partners. No significant differences were found across gender and origin.

Overall, 4.3% (*n* = 50) of participants admitted having a STI in the previous year: 5.1% (*n* = 31) of women and 3.5% (*n* = 19) of men; 6.1% (*n* = 24) of Africans, 4.0% (*n* = 16) of Brazilians and 2.8% (*n* = 10) of Eastern Europeans.

### 3.3. Use of Health Services and Preventive Initiatives

Table 2 also shows the distribution of ever use of health services, attendance to a sexual and reproductive health consultation and free condoms received by gender. A significantly higher proportion of women reported having ever used the health services and having attended a sexual and reproductive health consultation, while men reported more frequently having received free condoms in the previous year. Participants who ever used health services reported more frequently having been tested for HIV compared to those who never used (64.5% *vs.* 52.7%, *p* = 0.001). Similar result was found for attending a sexual and reproductive health consultation (74.7% among those who had attended *vs.* 58.6% of those who never attended, *p* < 0.001) (data not shown).

### 3.4. Factors Associated with no Condom Use in the Last Sexual Intercourse

Logistic regression estimation results for factors associated with the likelihood of not having used condom in the last sexual intercourse are presented in Table 3. After adjusting the logistic regression model to all variables presented in the analysis, having not used condom was more likely among women (OR = 1.73, 95% CI: 1.25–2.40), Africans (OR = 1.81, 95% CI: 1.11–2.96) compared to Eastern Europeans, those older (OR = 1.84, 95% CI: 1.02–3.32), those with elementary education (OR = 1.84, 95% CI: 1.11–3.01) compared to higher education, those married (OR = 2.38, 95% CI: 1.67–3.38) and those who didn’t receive free condoms in the previous year (OR = 1.49, 95% CI: 1.06–2.09). No condom use was less likely among immigrants who had only occasional sexual partners (OR = 0.10, 95% CI: 0.05–0.20) and both regular and occasional sexual partners (OR = 0.43, 95% CI: 0.25–0.75).

**Table 3:** Logistic regression results for factors associated with no condom use in the last sexual intercourse.

	*n* (%) (*n* = 635)	Crude OR (95% CI)	Adjusted OR (95% CI) ^1^
Sex			
Male	254 (54.2)	1.00	1.00
Female	381 (72.8)	2.27 (1.74–2.96) ^***^	1.73 (1.25–2.40) ^**^
Origin			
Eastern European	184 (64.3)	1.00	1.00
Brazilian	215 (57.5)	0.75 (0.55–1.03)	1.05 (0.68–1.61)
African	236 (71.1)	1.36 (0.97–1.91)	1.81 (1.11–2.96) ^*^
Age (years)			
18–24	78 (50.0)	1.00	1.00
25–44	418 (63.5)	1.74 (1.23–2.48) ^**^	1.20 (0.77–1.87)
45–65	139 (78.1)	3.56 (2.22–5.73) ^***^	1.84 (1.02–3.32) ^*^
Educational level			
Higher	206 (61.9)	1.00	1.00
Secondary	235 (59.0)	0.89 (0.66–1.20)	1.40 (0.95–2.07)
Elementary	191 (74.3)	1.78 (1.25–2.55) ^**^	1.84 (1.11–3.01) ^*^
Marital status			
Unmarried	301 (52.8)	1.00	1.00
Married	332 (79.0)	3.37 (2.53–4.49) ^***^	2.38 (1.67–3.38) ^***^
Number of sexual partners			
1 partner	447 (75.4)	1.00	1.00
2–3 partners	138 (54.3)	0.39 (0.29–0.53) ^***^	0.94 (0.60–1.48)
≥4 partners	31 (27.7)	0.13 (0.08–0.20) ^***^	0.76 (0.38–1.50)
Type of sexual partners in the previous 12 months			
Only regular	546 (75.6)	1.00	1.00
Only occasional	14 (14.1)	0.05 (0.03–0.10) ^***^	0.10 (0.05–0.20) ^***^
Regular and occasional	71 (44.7)	0.26 (0.18–0.37) ^***^	0.43 (0.25–0.75) ^**^
Free condoms received in the previous 12 months			
Yes	184 (51.8)	1.00	1.00
No	451 (70.8)	2.25 (1.72–2.95) ^***^	1.49 (1.06–2.09) ^**^
Ever testing for HIV			
Yes	410 (62.6)	1.00	1.00
No	225 (66.8)	1.20 (0.91–1.58)	1.13 (0.80–1.59)

^1^ Adjusted for sex, origin, age, educational level, marital status, number of sexual partners, type of sexual partners in the previous 12 months, free condoms received in the previous 12 months and ever testing for HIV. ^*^ *p* < 0.05; ^**^ *p* < 0.01; ^***^ *p* < 0.001.

## 4. Discussion

In general, the results of this study indicate high rates of risky sexual behaviors among immigrants, including having multiple (concurrent) sexual partners and unprotected sexual intercourse, consistently with other research conducted in Europe [6,31] and in other regions [9,29,32,33]. These findings highlight the potential increasing risk for HIV infection of migrants who have unprotected sex with multiple sexual partners, but also if they acquire HIV/AIDS the increasing likelihood of transmitting HIV infection to their partners.

As would be expected, we found wide variability in sexual risk behaviors across gender. Women reported significantly less condom use than men. This can be strongly related to gender power inequalities and lack of negotiating capacity to request safer sex, which renders women particularly vulnerable to partners’ risk behaviors [34,35]. On the other hand, although consistent condom use was more frequent among men, the reported higher rates of multiple sexual partners, engagement in commercial sex and with same-sex partners puts this subgroup at increased risk for HIV infection. A key element to be explored in future research is the variations in the pattern of risk practices through the different phases of the migration process and within each geographic context.

The overall reported HIV prevalence in this study was 2% and 4.4% in the African subgroup, much higher compared to that estimated for the general national population (0.7% among individuals aged 15–49 years) [17]. The scant published research in the European context show relatively great levels of infection. In a cross-sectional survey conducted in London with a convenience sample of Central and Eastern European migrants, 1.1% of respondents reported being HIV positive [6]. A study carried out in HIV counselling and testing clinics in several Spanish cities included a sample of immigrants who voluntarily tested for HIV and the HIV prevalence obtained ranged from 1.7% in Central and Eastern Europeans and in Latin Americans, 2.4% in North Africans and 8.4% in Sub-Saharan Africans [36]. In other cross-sectional community-based survey conducted with 1006 black-Africans in England, 14% of participants tested HIV positive [30]. Given that our data is solely based on self-reported information with absence of biological samples, we can speculate that the proportion of HIV-positive immigrants among our sample is probably higher.

The greater prevalence of HIV among African immigrants found in our sample has also been described in other European studies [30,36,37,38]. This certainly can be related to the higher prevalence of HIV in their countries of origin but also to their greater exposure to HIV risk. Indeed, African immigrants reported higher rates of risk sexual behaviors (multiple sexual partners, concomitant partners in the last year and inconsistent condom use-from further analysis not shown). In future research it is essential to further explore the patterns of risk behaviors of migrants and the underlying individual, sociocultural and contextual factors as also the epidemiological background.

A relevant result in our study is the observation of risky sexual behaviors among HIV-positive immigrants. Although caution is needed when analyzing the results because the sample size is small, a relatively large proportion of these participants reported having multiple sexual partners, having both regular and occasional partners in the previous year and engaging in unprotected sex, which poses direct risks for HIV transmission. Secondary prevention strategies are warranted to foster safer sex practices among those living with HIV.

It is also important to point out the high reported prevalence of STI found in this study. There is clear evidence that STI increase the likelihood of HIV transmission and similar sexual behaviors place people at high risk of both infections [39]. Our findings reinforce the need of strengthening integrated HIV and STI prevention and control programs among immigrants.

Approximately 38% of immigrants in this study reported having never been tested for HIV and of those who ever tested, 63.4% were not tested in the last year. These may include a potential group of immigrants with undiagnosed HIV infection. Moreover, immigrants who never tested for HIV reported more frequently risky sexual behavior. Evidence gained in continued experience in HIV testing indicates that increasing individuals’ awareness of HIV infection and counseling them to develop appropriate sexual protective measures leads to substantial reductions in high-risk sexual behavior [40,41,42]. Therefore, promotion of HIV testing among this population should be sustained.

When promoting HIV testing it must be taken into account the important finding observed in this study and in other research that having never been tested for HIV is significantly associated with having never used health services or attended a sexual reproductive health consultation [43,44]. Our results show a considerable proportion of immigrants who never used the health services, in line with previous studies [45,46,47,48]. Although no specific data were collected on barriers in utilization of health services, the literature points out migrants’ lack of knowledge on the health services available, the lack of cultural competence of health professionals and structural barriers as cost, distance of health services and legal restrictions [12,46,49,50]. These findings support the need to overcome barriers and promote the use of health services in order to reduce missed opportunities for early detection of HIV and prevention among this population.

Immigrants who received free condoms were less likely to had unprotected sex, suggesting that the provision of free condoms to other immigrants can have the same benefits. Additionally, we found a relationship between low educational level and having not used condom in the last sexual intercourse. A possible explanation is that lower educational level, frequently related to the lack of knowledge on HIV transmission and prevention, may hinder risk perception and increase the adoption of unsafe sexual practices [8,25,51,52]. Community-based initiatives promoted by outreach teams who have trusting relationships with the community and have unique knowledge of its context can contribute to promote effective HIV prevention interventions among these populations [10,53].

The findings must be interpreted with consideration of some limitations. The cross-sectional nature of the data limits our ability to draw causal inferences. Another limitation relates to the sampling procedure. Although the study achieved a diverse and large sample we did not use a random sampling method to select the study population. In fact, the information available on immigrant population in Portugal does not allow constructing sampling frames for representative population-based surveys that capture hard-to-reach subgroups as undocumented and recent immigrants. Collecting data only from migrants presenting themselves at the NISC potentially leads to an overrepresentation of more affluent and better integrated migrant groups. However, as this center is viewed by immigrants as an independent institution dedicated to solving individual integration problems, we are confident that the sampling procedure allowed for a fairly representative sample of migrant conditions. Also, the participants’ socio-demographic profiles are in line with data on immigrant populations in Portugal [19]. We did not collect data on reasons for refusals, but the topic explored in this study may have motivated individuals to refuse, which could mask a higher prevalence of risk behaviors and infection among this population. As in many other investigations, another limitation is the collected information based on self-reports. Due to the intimate and sensitive nature of the study subject and the high degree of stigma and discrimination attached to risky sexual behavior and HIV/STI positive serostatus, response bias toward under-reporting in this study would be expected.

Despite study limitations, this large-scale study provides useful insights on risky sexual behavior and HIV infection/STI among immigrants. In order to improve our understanding of the complex dynamics of HIV risk and infection in this population further research is needed.

## 5. Conclusions

In summary, this study shows that a considerable proportion of immigrants engage in risky sexual behaviors rendering them at high risk for HIV infection. Our findings highlight that efforts to promote HIV testing and awareness of HIV serostatus among immigrants as well as culturally adapted interventions promoting risk reduction strategies should be strengthened. Given the enormous heterogeneity of immigrant communities, tailored interventions should be developed targeting subgroups prone to high risk sexual behaviors and matching their specific prevention needs.
